# Long-term effectiveness of initiating non-nucleoside reverse transcriptase inhibitor- versus ritonavir-boosted protease inhibitor-based antiretroviral therapy: implications for first-line therapy choice in resource-limited settings

**DOI:** 10.7448/IAS.19.1.20978

**Published:** 2016-08-05

**Authors:** Viviane D Lima, Mark Hull, David McVea, William Chau, P Richard Harrigan, Julio SG Montaner

**Affiliations:** 1 British Columbia Centre for Excellence in HIV/AIDS, Vancouver, British Columbia, Canada; 2 Division of AIDS, Department of Medicine, Faculty of Medicine, University of British Columbia, Vancouver, British Columbia, Canada; 3 Public Health & Preventative Medicine Residency Program, University of British Columbia, Vancouver, British Columbia, Canada

**Keywords:** antiretroviral therapy, viral suppression, viral failure, resistance, NNRTI boosted PI, cohort study, British Columbia

## Abstract

**Introduction:**

In many resource-limited settings, combination antiretroviral therapy (cART) failure is diagnosed clinically or immunologically. As such, there is a high likelihood that patients may stay on a virologically failing regimen for a substantial period of time. Here, we compared the long-term impact of initiating non-nucleoside reverse transcriptase inhibitor (NNRTI)- versus boosted protease inhibitor (bPI)-based cART in British Columbia (BC), Canada.

**Methods:**

We followed prospectively 3925 ART-naïve patients who started NNRTIs (*N*=1963, 50%) or bPIs (*N*=1962; 50%) from 1 January 2000 until 30 June 2013 in BC. At six months, we assessed whether patients virologically failed therapy (a plasma viral load (pVL) >50 copies/mL), and we stratified them based on the pVL at the time of failure ≤500 versus >500 copies/mL. We then followed these patients for another six months and calculated their probability of achieving subsequent viral suppression (pVL <50 copies/mL twice consecutively) and of developing drug resistance. These probabilities were adjusted for fixed and time-varying factors, including cART adherence.

**Results:**

At six months, virologic failure rates were 9.5 and 14.3 cases per 100 person-months for NNRTI and bPI initiators, respectively. NNRTI initiators who failed with a pVL ≤500 copies/mL had a 16% higher probability of achieving subsequent suppression at 12 months than bPI initiators (0.81 (25th–75th percentile 0.75–0.83) vs. 0.72 (0.61–0.75)). However, if failing NNRTI initiators had a pVL >500 copies/mL, they had a 20% lower probability of suppressing at 12 months than pVL-matched bPI initiators (0.37 (0.29–0.45) vs. 0.46 (0.38–0.54)). In terms of evolving HIV drug resistance, those who failed on NNRTI performed worse than bPI in all scenarios, especially if they failed with a viral load >500 copies/mL.

**Conclusions:**

Our results show that patients who virologically failed at six months on NNRTI and continued on the same regimen had a lower probability of subsequently achieving viral suppression and a higher chance of evolving HIV drug resistance. These results suggest that improving access to regular virologic monitoring is critically important, especially if NNRTI-based cART is to remain a preferred choice for first-line therapy in resource-limited settings.

## Introduction

HIV treatment options for initial combination antiretroviral therapy (cART) for therapy naïve patients continue to expand, with more than 30 available drugs and co-formulations [1]. The goal of cART is to achieve sustained suppression of viral replication as a means to decrease HIV-related morbidity and mortality [2] and secondarily prevent HIV transmission [2,3]. Recommendations for first-line therapy regimens vary between guidelines; however, there is growing consensus that the preferred options include triple drug combinations which contain an integrase inhibitor (INI), a non-nucleoside reverse transcriptase inhibitor (NNRTI) and a ritonavir-boosted protease inhibitor (bPI) [2,4,5]. Options are often restricted in resource-limited settings where preferred initial regimens currently include two nucleoside reverse transcriptase inhibitors (nRTI) plus an NNRTI [5].

NNRTIs have been shown to be highly effective in achieving short-term virologic suppression. However, NNRTIs have a relatively low genetic barrier to the emergence of HIV drug resistance [6–8]. As a result, it is very important that patients on these regimens be closely monitored to decrease their chances of being on a failing regimen for a long period of time, which is associated with further evolution of HIV drug resistance. This is a particular concern in resource-limited settings, where plasma viral load (pVL) monitoring may not be available, and therefore, immunologic and/or clinical criteria are the only means of diagnosing treatment failure, which are criteria with known poor sensitivity and specificity [5,9,10]. In contrast, bPI-based cART regimens are more expensive and have a higher pill burden, but they also have a relatively higher genetic barrier to the emergence of HIV drug resistance [2,11].

Several studies have documented superior efficacy of NNRTIs versus bPIs based on rates of short-term viral suppression [6,12–17]. However, the relevance of these findings to settings where pVL monitoring is not available has not been elucidated. In other words, the long-term consequences of remaining on a failing NNRTI- versus bPI-based cART have not been previously evaluated. Thus, in this study, we compared the long-term effectiveness of NNRTIs and bPIs among patients remaining on their initial failing regimens in the province of British Columbia (BC), Canada.

## Methods

### Data

Study data from eligible patients were extracted from the BC Centre for Excellence in HIV/AIDS (BC-CfE)'s Drug Treatment Program (DTP) monitoring and evaluation system. Since October 1992, the distribution of antiretrovirals in BC has been the responsibility of the DTP. HIV medical care and laboratory monitoring are fully subsidized (free, without co-payments or deductibles) to all people living with HIV/AIDS (PLWHA) residents of BC according to BC-CfE's HIV therapeutic guidelines, which have remained generally consistent with those put forward by the International AIDS Society-USA, currently known as the International Antiviral Society-USA (IAS-USA), since 1996 [2,18,19].

Eligible participants were cART naïve patients, ≥19 years old, enrolled between 1 January 2000 and 30 June 2013 and followed until no later than 30 June 2014. These patients started cART consisting of two nRTIs as backbone, plus either an NNRTI (efavirenz or nevirapine) or a bPI (ritonavir booster plus either lopinavir or atazanavir). They must also have had a CD4 count and pVL measurement within six months of the initial antiretroviral date. All pVL measurements in BC are centrally done at the St Paul's Hospital virology laboratory. Since the quantification range of pVL assays has evolved over time, for analytical purposes, we truncated our measurements to range from <50 (coded as 49) to >100,000 (coded as 100,010) copies/mL [20–23]. CD4 cell counts are measured by flow cytometry, followed by fluorescent monoclonal antibody analysis (Beckman Coulter, Inc., Mississauga, Ontario, Canada). The CD4 data come from different laboratories across BC, and, in our database, we capture >85% of all CD4 tests done in BC. HIV drug resistance genotyping is available, free of charge, to all BC residents at the BC-CfE virology laboratory on samples with pVL ≥250 copies/mL, upon physician request. Methods for HIV-1 RNA extraction and drug resistance analysis have been described in detail elsewhere [24]. Note that in BC, approximately 90% of patients have clade B subtype, and only a small number have other subtypes (mostly clades A and C subtypes).

Resistant samples were assigned to one of four classes based on a modification of the 2014 IAS-USA list of mutations [25]: 1) lamivudine/emtricitabine resistance (M184V/I); 2) any other NRTI resistance (41L, 62V, 65R, 67N, 69D or insertion, 70E/R, 74V, 75I, 77L, 115F, 116Y, 151M, 210W, 215F/Y or 219 E/Q); 3) any NNRTI resistance (100I, 101E/H/P, 103N, 106A/M, 108I, 138A/G/K/Q/R, 181C/I/V, 188C/H/L, 190A/S, 225H, 230L or 236L); and 4) any protease inhibitor (PI) resistance (30N, 32I, 33F, 46I/L, 47A/V, 48V, 50L/V, 54V/L/M, 58E, 74P, 76V, 82A/F/L/S/T, 84V, 88S or 90M).

Mortality data for all causes were obtained through monthly linkages with the BC Vital Statistics Agency. Patients lost to follow-up were censored at the last contact date (i.e. the date for a laboratory test, a prescription refill or a physician visit) prior to any of the following events: 1) if they moved outside of BC; 2) if last contact date was more than 18 months and before 31 December 2012; 3) if last contact date was less than 18 months and after 31 December 2012; 4) if they enrolled in a blinded trial involving receiving placebo medication; or 5) if they had a scheduled treatment interruption.

### Statistical methods

For the purpose of the analyses in this study, patients were included if they did not change cART regimen during the study period, and they were required to have a follow-up of at least six months. Thus, we followed patients since cART initiation, and at six months following cART initiation, we assessed whether patients virologically failed therapy, defined by a pVL >50 copies/mL. Based on the pVL at failure, failing patients were stratified at pVL ≤500 versus >500 copies/mL. We continued to follow those patients who failed at six months for an additional six months, on the same cART regimen, and calculated their probability of achieving subsequent viral suppression defined as a pVL <50 copies/mL twice consecutively (yes or no) and of developing drug resistance to any cART class of medications they were currently taking (yes or no), defined by any combination of the above resistance classes. For analytical purposes, samples with pVL <250 copies/mL were classified as wild type virus. We restricted this analysis to include patients with no resistance prior to starting cART.

These probabilities were modeled via a multivariable logistic regression explanatory model, separately for those who started on NNRTI-based and bPI-based cART. Possible explanatory variables for the viral suppression and resistance analyses at 12 months after cART initiation included age (continuous), gender (male or female), history of IDU (yes, no or unknown), CD4 cell count at baseline and at six months (continuous), pVL at six months (≤500 or >500 copies/mL), period of cART initiation (2000–2005, 2006–2009 or 2010–2013), adherence level measured between six and twelve months following cART initiation (<95% or ≥95%) and follow-up time (in months). We did not include pVL measured at baseline since it is highly collinear with pVL measured at six months; otherwise, it may bias our coefficient's standard errors. Adherence level, for each period, was defined as the number of days of antiretroviral drugs dispensed divided by the number of days of follow-up (expressed as percent). Estimates of adherence to antiretroviral therapy were based on the different regimen exposures for each patient. Although patients with undetectable pVL less commonly have medications changed, the regimens can change due to adverse effects or other medical and non-medical reasons, and we also accounted for those changes. Measuring adherence to any specific antiretroviral drug as index medication when it can be changed at any point in time makes it difficult for accurate monitoring. For this reason, the measure of adherence that we chose to adopt does not use any specific index medication, and it only measures the overall exposure to any antiretroviral regimen [26]. Although currently antiretroviral regimens may not need adherence levels ≥95% to achieve viral suppression in the short and long terms, this cut-off is still highly relevant based on the regimens used during our study period and also since drug resistance was one of our outcomes. Previous studies, including patients on NNRTIs or boosted bPI, have indicated that adherence levels between 80 and 95% are associated with the highest likelihood of acquiring drug resistance [27]. Follow-up time for viral suppression, between six and twelve months, was measured until the date patients experienced the outcome or until the last contact date or date of death if they did not experience the outcome during this six-month period. The follow-up time for the development of resistance followed the same rational.

For building multivariable models, a modified backward stepwise technique, based on Akaike Information Criterion (AIC) and Type III *p*-values, was used in the selection of explanatory variables [26]. Goodness of fit was assessed using the area under the receiver operating characteristic curve to measure the model's ability to discriminate between those who achieved the outcome and those who did not. Categorical variables were compared using the Fisher's exact test (for 2×2 tables) or the Cochran–Mantel–Haenszel test (for other table sizes), and continuous variables were compared using the Kruskal–Wallis test [28]. Rates were calculated dividing the number of events by the number of person-months of follow-up; corresponding 95% confidence intervals for these rates were based on the Fisher's exact test [28]. All analyses were performed using SAS version 9.4 (SAS, Cary, NC).

The BC-CFE received approval for this study from the University of British Columbia ethics review committee at the St Paul's Hospital, Providence Health Care site (H05–50123). The study complies with the BC's Freedom of Information and Protection of Privacy Act. The study was conducted using anonymized administrative databases, and therefore, informed consent was not required.

## Results

Among 3925 individuals who started cART between 1 January 2000 and 30 June 2013 and were followed until 30 June 2014, 1963 (50%) individuals started therapy on an NNRTI-based regimen, 1962 (50%) individuals started therapy on a bPI-based regimen, 81% were males, 45% did not have a history of injection drug use at baseline, 31% started cART in 2000 to 2005, 38% in 2006 to 2009 and 31% in 2010 to 2013. At baseline, the median age was 42 years (25th–75th percentiles (Q1–Q3 35–49), median CD4 cell count was 220 (Q1–Q3 130–340) and median pVL was 4.88 log_10_ copies/mL (Q1–Q3 4.36–5.00).

The number of patients who remained on the initial cART regimen within the first 12 months of follow-up was 3359 (86% of 3925). This consisted of 1616 (82% of 1963) in the NNRTI cohort and 1743 (89% of 1962) in the bPI cohort. At six months, we observed that 401 (25% of 1616; 9.5 cases per 100 person-months) patients in the NNRTI cohort and 601 (34% of 1743; 14.3 cases per 100 person-months) in the bPI cohort were classified as having virologic failure (i.e. a pVL >50 copies/mL) (*p* <0.0001 for the comparison of these two rates). Table 1 stratify these patients by whether they failed with a pVL ≤500 or >500 copies/mL. In both cohorts, patients failing with a high pVL (>500 copies/mL) were more likely to be female, to have a history of injection drug use at baseline, to be younger, to have lower baseline CD4 cell count and to have lower CD4 cell recovery during the first six months of follow-up (*p* <0.05).

**Table 1:** Patient characteristics stratified by initial combination antiretroviral therapy and viral load at therapy failure at six months, in British Columbia, 2000–2014

	Initial NNRTI-based regimen			Initial boosted PI-based regimen		
				
	Viral load at therapy failure at 6 months			Viral load at therapy failure at 6 months		
				
	≤500 copies/mL	>500 copies/mL		≤500 copies/mL	>500 copies/mL	
				
Variables	(*N*=243)	(*N*=158)	*p*	(*N*=460)	(*N*=141)	*p*
Gender, *n*(%)			0.0082			0.0153
Male	203 (64%)	114 (36%)		380 (79%)	103 (21%)	
Female	40 (48%)	44 (52%)		80 (68%)	38 (32%)	
History of injection drug use, *n*(%)			<0.0001			<0.0001
No	106 (73%)	40 (27%)		244 (86%)	40 (14%)	
Yes	87 (48%)	96 (52%)		154 (63%)	90 (37%)	
Unknown	50 (69%)	22 (31%)		62 (85%)	11 (15%)	
cART era, *n*(%)			<0.0001			0.8077
2000–2005	74 (46%)	87 (54%)		116 (75%)	39 (25%)	
2006–2009	76 (65%)	41 (35%)		200 (77%)	61 (23%)	
2010–2013	93 (76%)	30 (24%)		144 (78%)	41 (22%)	
cART adherence from 6 to 12 months of follow-up, *n*(%)			<0.0001			<0.0001
≥ 95%	165 (72%)	64 (28%)		331 (82%)	75 (18%)	
< 95%	73 (45%)	90 (55%)		121 (65%)	64 (35%)	
Age (years), median (Q1–Q3)	43 (37–51)	40 (33–47)	0.0006	42 (36–50)	41 (35–48)	0.0440
CD4 cell count (cells/mm^3^), median (Q1–Q3)						
At cART initiation	210 (120–340)	170 (80–290)	0.0248	170 (80–290)	170 (80–240)	0.2945
Change from cART initiation to six months of follow-up	120 (60–220)	40 (-10–135)	<0.0001	140 (70–240)	60 (0–160)	<0.0001
Change from cART initiation to 12 months of follow-up	160 (80–260)	80 (0–170)	<0.0001	190 (110–305)	80 (0–210)	<0.0001
Viral load at ART initiation (log_10_ copies/mL), median (Q1–Q3)	5.00 (4.78–5.00)	5.00 (4.60–5.00)	0.2906	5.00 (4.88–5.00)	5.00 (4.65–5.00)	0.0253
Follow-up from baseline to 12 months (months), median (Q1–Q3)	10.6 (9.8–11.3)	10.7 (9.5–11.4)	0.9636	10. 8 (9.8–11.4)	10.4 (9.5–11.2)	0.1935

cART, combination antiretroviral therapy; NNRTI, non-nucleoside reverse transcriptase inhibitors; PI, protease inhibitors; Q1, 25th percentile; Q3, 75th percentile.

These patients continued to be followed for another six months, in the same initial cART regimen, in which we assessed whether they subsequently achieved viral suppression and/or developed drug resistance to any medication they were currently taking. During this time, in both cohorts, patients who failed with a high pVL (>500 copies/mL) at six months were more likely to have lower adherence (<95%) and they continued to have lower CD4 cell recovery (*p* <0.0001) (Table 1).

Table 2 shows the patient outcomes at 12 months, among those who remained on the same cART regimen since treatment initiation. We observed that suppression rates at 12 months in the NNRTI cohort, for those who failed at six months with a pVL >500 copies/mL, were 68% lower than those for patients who failed with a pVL ≤500 copies/mL (1.6 vs. 5.0 cases per 100 person-months), while in the bPI cohort these rates were 80% lower (1.1 vs. 5.4 cases per 100 person-months). Note that these rates were in the same order of magnitude for both cohorts (no statistical difference). In this same table, we also present the patient outcomes in terms of development of resistance to any ART class. We observed that resistance rates at 12 months in the NNRTI cohort, for those who failed at six months with a pVL **≤**500 copies/mL, were 85% lower than those for patients who failed with a pVL **>**500 copies/mL (0.2 vs. 1.3 cases per 100 person-months), while in the bPI cohort these rates were 67% lower (0.1 vs. 0.3 cases per 100 person-months). Furthermore, note that the resistance rates for those in the bPI cohort were significantly lower than the same rates for the NNRTI cohort for patients with a pVL **>**500 copies/mL at six months (*p*<0.0001), and these rates were in the same order of magnitude for both cohorts among patients with a pVL **≤**500 copies/mL at six months (no statistical difference).

**Table 2:** Patient outcomes at 12 months stratified by initial combination antiretroviral therapy and viral load at therapy failure at six months, in British Columbia, 2000–2014

	Initial NNRTI-based regimen			Initial boosted PI-based regimen		
				
	Viral load at therapy failure at 6 months			Viral load at therapy failure at 6 months		
				
	≤500 copies/mL	>500 copies/mL		≤500 copies/mL	>500 copies/mL	
				
Variables	(*N*=243)	(*N*=158)	*p*	(*N*=460)	(*N*=141)	*p*
Viral suppression at 12 months, *n*(%)						
No	48 (20%)	94 (60%)	<0.0001	140 (31%)	76 (55%)	<0.0001
Yes	190 (80%)	60 (40%)		312 (69%)	63 (45%)	
Rate per 100 person-months (95% Confidence Interval)	5.0 (4.3–5.7)	1.6 (1.2–2.0)	<0.0001	5.4 (4.8–6.0)	1.1 (0.8–1.4)	<0.0001
Development of resistance to any ART class at 12 months, *n*(%)						
No	165 (96%)	56 (64%)	<0.0001	321 (98%)	72 (87%)	<0.0001
Yes	6 (4%)	31 (36%)		5 (2%)	11 (13%)	
Rate per 100 person-months (95% Confidence Interval)	0.2 (0.1–0.5)	1.3 (0.9–1.8)	<0.0001	0.1 (0.1–0.3)	0.3 (0.1–0.5)	0.2101

ART, antiretroviral; NNRTI, non-nucleoside reverse transcriptase inhibitors; PI, protease inhibitors.

**Figure 1:**
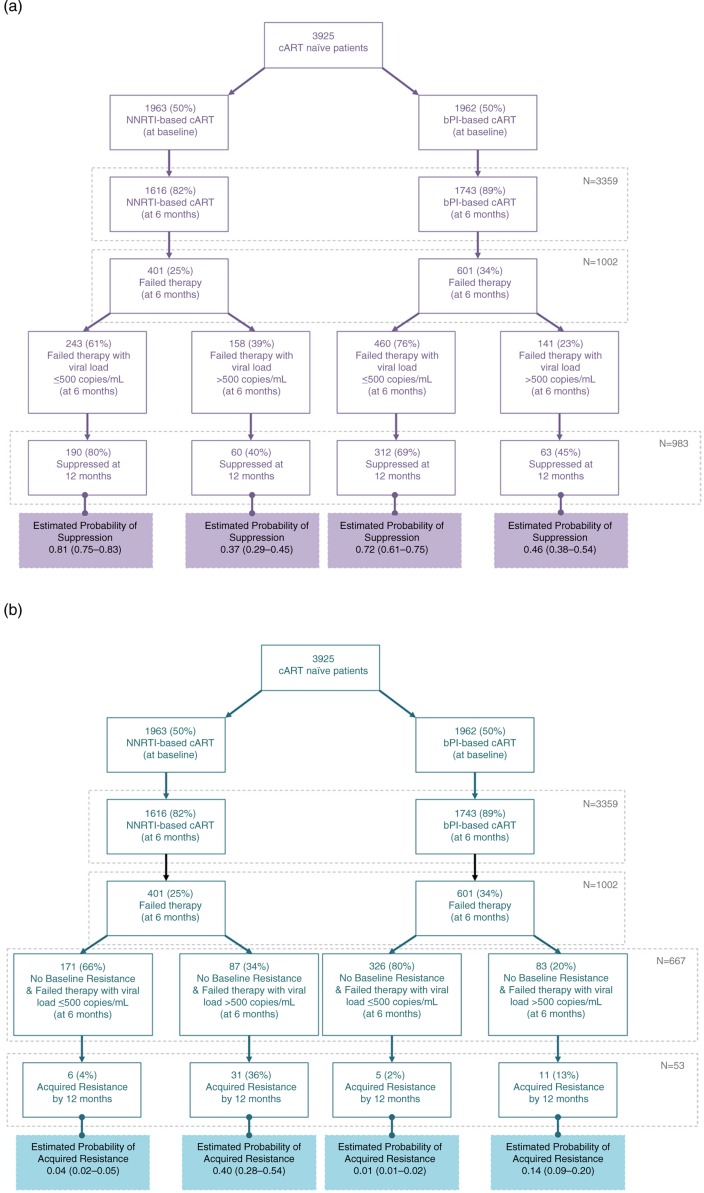
Study patient flow and estimated probability of viral suppression and of developing ART drug resistance at 12 months stratified by initial combination antiretroviral therapy and viral load at therapy failure at six months, in British Columbia, 2000–2014. Estimated probabilities are expressed as median (25th–75th percentiles). (a) Viral suppression and (b) ART drug resistance.

Figure 1 shows the estimated probabilities from the multivariable models for both outcomes. Patients on NNRTI-based regimens, who failed therapy with a pVL ≤500 copies/mL at six months, had a 11% higher probability of achieving subsequent suppression at 12 months than those on bPI (median (Q1–Q3): 0.81 (0.75–0.83) vs. 0.72 (0.61–0.75); *p* <0.0001). However, if patients on NNRTI-based regimens failed therapy with a pVL >500 copies/mL at six months, they had a 20% lower probability of suppressing at 12 months than those on bPI (0.37 (0.29–0.45) vs. 0.46 (0.38–0.54); *p* <0.0001). In terms of resistance, those who failed on NNRTI performed worse than bPI in all scenarios, especially if they failed at six months with a pVL >500 copies/mL (0.40 (0.28–0.54) for NNRTI vs. 0.14 (0.09–0.20) for bPI; *p* <0.0001).

## Discussion

Our results illustrate the different impact of remaining on a virologically failing regimen depending on whether it was an NNRTI- or a bPI-based cART. Patients who failed with a high pVL and continued on the first-line NNRTI regimen had a lower probability than those on bPI with regard to the likelihood of subsequently achieving viral suppression, and a higher chance of experiencing evolving HIV drug resistance, in all scenarios. Our results, therefore, suggest that in the absence of pVL monitoring, using NNRTI-based cART regimens as first-line will result in an unintended excess HIV drug resistance prevalence and compromise second-line therapy regimens. Also of concern, the NNRTI-resistant variants have a relatively good fitness and, thus, are likely to be the source of transmitted HIV drug resistance [29].

These results are particularly relevant as we move towards the implementation of the 2015 WHO guidelines recommending universal access to cART for all PLWH regardless of CD4 cell count levels, globally [30]. These guidelines were based on the overwhelming evidence for the efficacy of early treatment [31,32], and its release helped the Joint United Nations Programme on HIV/AIDS (UNAIDS) 90-90-90 Target gain substantial momentum as means to eliminate the AIDS pandemic [33]. While it may be tempting to suggest that replacing NNRTI-based cART as first-line therapy could be a way to address this issue, we feel that this would be challenging in resource-limited settings, and it would fail to address the fundamental need of optimizing treatment monitoring in this setting. Indeed, we interpret our findings as highlighting the critical and urgent need to secure access to pVL monitoring globally [34].

Unfortunately, routine pVL monitoring remains unavailable in many resource-limited settings [5,25]. This is despite the well-established fact that cART treatment switching guidelines based on clinical or CD4 criteria have low sensitivity and specificity [35,36]. Our findings further emphasize that remaining on a failing cART regimen has serious implications and may compromise virologic response to subsequent regimens. As such, a renewed effort is needed to optimize access to pVL monitoring globally, including in resource-limited settings [13,36,37]. Encouragingly, on 25 September 2014, a lower cost pVL assay was announced at a side event during the United Nations General Assembly [38], which can be used to properly monitor these patients.

There are some features of this study that are worth mentioning. The study data were based on patient data within a population-based HIV treatment programme with free access to medical care, antiretroviral therapy and laboratory monitoring. Second, estimates of adherence (pharmacy refill record) to antiretroviral therapy were based on the medication exposure of each patient, which is the maximum adherence an individual might have during a period of time. Currently, there is no gold standard to measure adherence to cART. However, this type of adherence measure was found to be highly predictive of disease progression and death, among other key clinical outcomes [39]. Third, this study was based on individuals naïve to cART, thus making our results not influenced by confounding previous therapy use. Fourth, some might argue that our outcome definitions could have played a role in explaining these trends; however, our sample size remained sufficiently large to conduct these analyses. Fifth, through our DTP we have precise longitudinal information on the type of regimen each of our patients receive, thus allowing us to conduct this study by eliminating any bias that could have been introduced due to therapy switches. Sixth, one could argue that this study could have been influenced by confounder by indication, since initial cART is not assigned at random. However, since we stratified our analyses by the type of regimen received at baseline, we controlled for this source of bias. Seventh, it is important to acknowledge that there are differences in patient characteristics between BC and resource-limited settings (e.g. the prevalence of injection drug use, adherence levels and gender distribution), and we did not mean to imply that these two settings are similar. However, we believe that if patients in both of these settings are offered the same ART regimens, biologically, there is no difference in how they will respond to therapy. Eighth, in BC, in comparison with resource-limited settings, tuberculosis co-infection is relatively rare [40,41]. Although there is extensive documentation regarding the interaction between different tuberculosis and HIV medications, antiretroviral treatment outcomes are highly heterogeneous among co-infected individuals and highly dependent on treatment adherence. A recent meta-analysis including only resource-limited settings studies showed no difference in virologic failure/success comparing co-infected and HIV mono-infected patients [42]. Thus, given that all our analyses were adjusted for HIV treatment adherence and tuberculosis is relatively rare in our population, we believe that the differences observed between the group of patients receiving either NNRTI or boosted PI were not due to HIV/tuberculosis co-infection. Finally, although we adjusted our analyses for several demographic and clinical characteristics, as in all observational studies unmeasured differences may exist among study populations, and for this reason, our findings should be interpreted cautiously. Additionally, given its observational nature, this study served to compare the effectiveness of NNRTIs and boosted PIs, while efficacy of these regimens were previously demonstrated using randomized designed studies such as in the “EARNEST trial” [43].

## Conclusions

Our results showed that NNRTI-based cART had superior virologic short-term effectiveness than bPI-based cART. In contrast, among those patients who failed with a high pVL (≥500 copies/mL) at six months, NNRTI starters were less likely to achieve subsequent virologic suppression and had a higher probability of developing HIV drug resistance. These results highlight the critical and urgent need to secure global access to pVL monitoring.
